# The relationship between social capital and health from a configuration perspective: an evidence from China

**DOI:** 10.1186/s12889-023-16547-1

**Published:** 2023-08-24

**Authors:** Chongqi Hao, Dan Guo, Hao Ren, Xuchun Wang, Yuchao Qiao, Lixia Qiu

**Affiliations:** 1 School of Public Health, Shanxi Medical University, Taiyuan, China; 2 School of Management, Shanxi Medical University, Taiyuan, China

**Keywords:** Social capital, Health, FsQCA, CFPS, Configuration analysis

## Abstract

**Background:**

The debate on the relationship between social capital and health is still ongoing. To enhance previous research, this study uses data drawn from China to analyse the situations in which social capital is related to good health and the various configurations that result in good health outcomes.

**Methods:**

Using the data of China Family Panel Studies, the conditions of age, gender, marriage, education, income, structural social capital and cognitive social capital were included to analyse the sufficient and necessary conditions for achieving good general health and their different configurations using the fsQCA method.

**Results:**

None of the listed conditions were prerequisites for excellent general health in terms of either their presence or their absence. The sufficiency analysis found 11 configurations with an average of 3–4 conditions per configuration; in no configuration was the condition of social capital present alone. Structured social capital and cognitive social capital exhibited negative states in configurations 1 and 2, respectively. The most prevalent factor in all configurations was the condition of age.

**Conclusions:**

The relationship between social capital and health is both positive and negative, with cognitive social capital playing a larger role in the positive relationship than structural social capital. Social capital is neither a necessary nor a sufficient condition for health, and it must be combined with a variety of other factors to promote health. A variety of methods can be used to promote an individual's health, as different populations require different approaches to good general health, and no single pathway applies to all populations. In the Chinese population, an individual's age is a significant determinant of their health status.

## Background

After more than 20 years of research, the debate on the relationship between social capital and health is still ongoing. Research on this topic is challenging since social capital is a complicated concept and health measurements are multidimensional. A large body of empirical research has suggested that social capital has a positive impact on health, while some studies have suggested that its impact is weak; in addition, some evidence has shown that these two variables are not statistically associated or even negatively correlated. Many empirical studies have provided a rich empirical basis for studying the relationship between social capital and health by using linear regression models, in which context the variables are assumed to be independent of each other and the model is symmetric; however, the relationship between social capital and health may depend on one other condition or a combination of such conditions, and the absence of certain conditions may not affect the achievement of positive health outcomes, an issue which is clearly difficult to address in linear regression models. The approach of qualitative comparative analysis, which has been widely used in management science, can be used to analyse the relationships among multiple variables and outcomes from a holistic perspective, and more insights may be gained by applying this approach to the study of the relationship between social capital and health.

A study employing a qualitative comparative analysis approach was used to investigate the link between social capital and health, proving the method's viability. However, the applicability of this study is limited because it was an exploratory study based on data drawn from the European Social Survey and did not take marriage and education into account [1]. To explore the relationship between social capital and health in further detail from a configuration perspective, this study uses data drawn from the China Family Panel Studies (CFPS), incorporates multiple conditions, such as marriage and education, and applies the fsQCA method. As a result, it offers evidence from China to support research in this field.

### The conception of social capital

The concept and theory of social capital have been some of the most controversial in the social sciences over the past 20 years. Such controversy has arisen primarily as a result of inconsistencies in the various definitions used for this concept. Bourdieu and Coleman are regarded as the originators of the theory of social capital. Bourdieu viewed social capital as a resource that is constructed by individuals based on their connections with others [2]; this view is essentially a definition of social capital from the perspective of social networks. In contrast, Coleman was more concerned with understanding and defining social capital at the group level and from a social integration perspective, drawing on results in the field of economic sociology and suggesting that "social capital is defined by its function" [3]. Social capital is composed of a variety of different entities, which share two common elements: they all consist of some aspect of social structures, and they facilitate certain actions on the part of actors, whether individual persons or corporate actors, within the structure. Putnam then further distinguished among the social trust, social norms, and social network measurement dimensions of social capital from a social integration perspective and identified the various types of social capital as structural social capital, cognitive social capital, bridging social capital and bonding social capital [4]. Putnam's definition and operationalization of social capital had a significant influence on subsequent research on social capital and health. Since that time, the social network school and the social integration school have taken initial shape, and subsequent definitions of social capital have largely not progressed significantly beyond this level.

In the study of social capital, the commonly used classifications are cognitive social capital and structural social capital, which have played an important role in guiding subsequent research [5]. Cognitive social capital refers to people's perceptions of the level of interpersonal trust and norms of reciprocity within a group and encompasses aspects of trust, solidarity, and reciprocity; in this context, the core component is trust, which can be further categorized into broad and specific trust [6, 7]. Broad trust denotes trust in unknown others, while specific trust denotes trust in known others [8]. Structural social capital refers to the formal or informal opportunity structures that individuals need to structure their social networks and relationships; it represents the participatory behaviour and engagement of individuals within a network that can be observed externally, encompassing social networks, civic activities, organizational memberships, etc [9–11]. Structural social capital can be divided into the micro level, where the focus is on the individual's participation in and the benefits received from social networks [12], and the macro level, which refers to the individual's opportunities to participate in social activities, for example, through a variety of associations [7]. There are clear differences between the two types of social capital. First, structural social capital is more formal than cognitive social capital; second, the form of cognitive social capital is subjective and more difficult to measure than the objective form of structural social capital. In empirical analyses, measures of structural social capital typically include social ties [13], network size and diversity [14], information channels and ethical infrastructure, and neutrality [15]. The most important indicator of cognitive social capital is trust, followed by shared goals and common culture, a common language, etc [16].

Two additional commonly used dimensions of social capital are bridging social capital and chain social capital. Bridging social capital refers to outwards-looking horizontal social ties among members of a heterogeneous network, which are usually directed towards strangers, members of ordinary associations, people with whom one is less familiar, and other general relations; these ties can be viewed as weak relationships that help individuals "get by" [17, 18]. Chain social capital can be viewed as a specific type of bridging social capital that refers to the social capital that results from heterogeneous, vertical ties associated with power differences or hierarchical differences [19, 20]. The development of social capital theory provides a solid theoretical foundation for contemporary research, and the present study also builds on this theoretical foundation.

### Social capital and health

Research on the relationship between social capital and health has been ongoing for more than twenty years. Since Kawachi et al. proposed this concept in their 1997 article "Social capital, income inequality, and mortality", social capital has been listed among the important social determinants of health [21, 22]. A systematic review published by Ehsan et al. in 2019 showed that a large number of studies have confirmed that social capital is significantly associated with positive health outcomes, outnumbering that have produced negative or nonsignificant results [23]. Xue et al. conducted a systematic review of the literature on the relationships among different types of social capital and multiple health outcomes and found that social capital is significantly associated with a variety of positive health outcomes but that this effect is very small and statistically nonsignificant [24]. This finding suggests that the pathways through which social capital affects health may be diverse and complex.

The pathways by which social capital impacts health are becoming clearer as empirical research continues to advance; accordingly, the following four specific pathways can be identified. The first such pathway is the social support pathway, which implies that social capital can provide material and emotional support, leading to significant physical and mental health benefits [25, 26]. If social capital refers to a social resource embedded in a social network or the ability and opportunity to access social resources through a social network, then one main way in which social capital operates is through access to social support as an important social resource [27]. Second, the lifestyle pathway, according to which social capital enhances individuals' health behaviours in terms of diet, exercise, and sleep, leads to better self-rated health, lower levels of depression, and greater psychological well-being [28]. The third pathway is the information dissemination pathway, according to which the social connectedness inherent in social capital allows health-related behaviours to spread through individuals' social networks through the transmission and diffusion of information or behavioural norms [29]. Finally, the fourth pathway is the social integration and control pathway, according to which higher levels of informal social control and collective efficacy in a society or group reflect better social integration, thus helping reduce adverse health behaviours and maintain good social functioning within the group [30].

## Methods

### Data sources

The data used in this study are drawn from the China Family Panel Studies(CFPS). The CFPS is conducted by Peking University's China Social Science Survey Centre. Individual, household, and community data are collected from 25 provinces/municipalities/autonomous regions. In the survey, residents are asked about their family relationships, living environment, and economic situation via a questionnaire. In addition, to comprehensively assess residents' health status, the survey evaluates their education, occupation, lifestyle, pension, and medical care [31]. The research findings contained in this database are reliable because the survey process is scientific and standardized, and the data quality is high [32]. The database has been updated every two years since 2010 and is now up to date through 2020.

The data used in this study are drawn from the 2020 CFPS individual pool, which contains a total of 28,590 records. First, we selected individuals aged 16 years or older (age >  = 16), and then we selected individuals who received a self-response questionnaire (selfrpt = yes) and completed the interview (iinterv = has completed). Finally, we excluded cases that reported "missing", "inapplicable", "invalid responses", "refusal to answer", or "don't know" for any of the variables used, resulting in a sample of 17,475 observations for the final analysis.

### Variable selection

The health indicator we used is "general health", which refers to self-rated health; this measure has been shown to be a reasonable indicator of an individual's true level of health [33], taking into account both the respondent's physical and mental health. It corresponds to the following question: "How would you rate your health status?"[QP201]. The response options are "1 excellent, 2 very good, 3 good, 4 fair, and 5 poor". Based on the theoretical summary provided above, we used the indicators of cognitive social capital and structural social capital to measure social capital. With regard to structural social capital, studies utilizing CFPS data have frequently used the term "gift spending" to reflect social networks [34–36]; however, this indicator is household-based and difficult to specify at the individual level. The CFPS 2020 individual questionnaire does not include questions pertaining to informal social connections; thus, we selected "interpersonal relationships" [37] as an indicator of structural social capital at the individual level. The question for "interpersonal relationships" is "Do you think you are popular?" (PM2011). The respondents were asked to rate this statement on a scale ranging from 0 to 10, with 0 being the lowest and 10 being the highest. With respect to cognitive social capital, we selected the commonly used indicator "general trust". This indicator has been used more frequently in other empirical studies [38–42]. The question for "general trust" is "In general, do you think that most people are trustworthy, or it is better to take greater caution when getting along with other people?" (PN1001). The response options were categorized as "Most people are trustworthy" and "The more caution, the better".

Other conditions we selected were "age", "gender", "education", "marriage" and "income", which have been included as control variables in the overwhelming majority of social capital and health studies and play a broad and important role in the pathway by which social capital impacts health [39, 43–46]. The condition of "age" indicates the age of the respondent in the year the survey was conducted and corresponds to the code "age", while "gender" corresponds to the code "gender". "Education" corresponds to the code "cfps2020edu", which indicates the highest level of education the respondent had completed in 2020 on a scale featuring 8 options, which were listed in the following order: "Illiterate or semiliterate, Primary school, Junior high school, High school/technical secondary school/technical school/vocational high school, Junior college, College degree, Master’s degree, and Doctorate". We chose "marriage-last" to represent the respondent’s most recent marital status, which is categorized as "Married (with a spouse), Divorced, Unmarried, Widowed, or Cohabiting". Given the reality of low self-reported income amounts, for the income profile, we selected job income satisfaction as a subjective indicator of income [47]; this measure corresponds to the question "How satisfied are you with your income from this job?" (QG401) This item was scored on a 5-point Likert scale ranging from very dissatisfied to very satisfied, with corresponding scores of 1–5.

### Model construction

We used fuzzy-set qualitative comparative analysis (fsQCA) to examine the sufficient and necessary conditions that lead to good general health as well as their various configurations. This study used the corresponding software fsQCA 4.1 for data analysis, following the form of presentation of results proposed by Ragin and Fiss [48] to organize the analysis results. QCA is a set-theoretic grouping analysis method based on Boolean algebra. Unlike traditional quantitative analysis, which is based on variance and the null hypothesis significance test, QCA treats cases as groups of variables and analyses the "necessary" or "sufficient" conditions for obtaining the desired outcome based on an identification of the particular outcome and variables to be explained, thereby holistically exploring “how” multiple concurrent causes and effects can generate complex problems [49]. QCA exhibits two crucial characteristics: equivalence and asymmetry. Equivalence suggests that "multiple combinations of antecedent conditions are equally effective", while asymmetry is interpreted as a situation in which "a condition (or a combination of conditions) that explains the presence of an outcome can be different from the conditions that lead to the absence of the same outcome" [50]. Based on these two properties, QCA can analyse different configurations of necessary and sufficient conditions for attaining the target outcome [51], which is not possible using a linear regression model.

QCA can be divided into 3 basic categories based on the type of variables: crisp-set qualitative comparative analysis (csQCA), multi-value qualitative comparative analysis (mvQCA), and fuzzy-set qualitative comparative analysis (fsQCA). csQCA analyses can address only binary categorical variables. mvQCA analyses superior for multicategorical nominal variables, but they are only suited to deal with the kind problem; cases can only be assigned to one of the categories associated with the categorical variables [52]. The emergence of fsQCA further enhances researchers’ ability to analyse fixed-distance and fixed-ratio variables, allowing QCA to address not only category problems but also degree-varying problems and partial membership, in which context cases have an affiliation score ranging between 0 (nonmembership) and 1 (full membership) [53]. Therefore, fsQCA was chosen for this study to facilitate the analysis of multiple variable types. As a methodological innovation, QCA aims to identify causal relationships among variable groupings and outcomes using case-to-case comparisons, thereby answering the question, "What groupings of variables lead to the desired outcome? " [54] QCA conceptualizes causality in terms of complex causation characterized by jointness, equivalence, and asymmetry. Combined with a group analysis approach, QCA researchers are able to extend the extant causal theoretical framework based on additivity and symmetry and revisit previous empirical findings and contradictory conclusions [55]. Using QCA analysis, researchers can also identify variable groupings of states with equifinality, thus improving their understanding of the differentiated driving mechanisms that lead to outcomes in different case scenarios and facilitating further discussion of the fit and substitution relationships among conditions. In addition, researchers can further compare the groupings of variables that lead to the results in question and broaden the dimensions of their theoretical explanations with regard to specific research questions [56].

QCA identify sufficient or necessary conditions by reference to affiliation between sets. A configuration is necessary if it is a consistent superset of the outcome; similarly, if the configuration is a consistent subset of the outcome, then the sufficiency of the configuration is indicated [57]. In crisp-set QCA, the boundaries are clearer, whereas in fuzzy-set QCA, the results must be judged using two metrics that vary between 0 and 1: consistency and coverage. For necessity analysis, the thresholds for the two indicators are typically 0.90 and 0.60, respectively [58], while the requirements for sufficiency analysis are relatively lenient, with consistency results greater than 0.80 being accepted [59].

Before performing fsQCA, the variables for the cause and effect conditions must be calibrated to fuzzy sets ranging from 0 and 1, with 0 denoting nonmembership and 1 denoting full membership. We used fsQCA software to complete the calibration process of the variables [60]. As suggested by a relevant study [61], the variables must be transformed into a calibrated set using three substantively meaningful thresholds: full membership, full nonmembership, and a crossover point that reflects maximum ambiguity. The dichotomy of health is more common, and in this context, we referred to several empirical studies that have placed the option 'fair' in the 'unhealthy' category [62, 63]; thus, we defined "excellent, very good, and good" as indicating full membership and "fair and poor" as indicating nonmembership. Next, we chose "general trust" as an indicator of cognitive social capital, and the possible responses for the question were treated as dichotomous by assigning "yes" to 1 and "no" to 0; scores of 1 and 0 were then classified as indicating full membership and nonmembership, respectively, without calibration. The structural social capital indicator took a score ranging from 0–10, for which we used the 90th, 10th, and 50th percentiles of the original distribution to define the thresholds and intersection points, which were calculated to be 10, 7, and 5, respectively. In the other conditions, age was calibrated in the same way as the structural social capital indicator, and gender was assigned a value of 1 for females and 0 for males to emphasize the analysis of females given that females are more likely to report poor health. With regard to education, we classified "junior college and college degree" as one category and then assigned a score ranging from 1–7, with 6 indicating full set membership, 4 indicating intermediate membership and 2 indicating full set nonmembership according to the 7-point Likert scale [49]. Income was calibrated on a 5-point Likert scale, with full set membership, intermediate membership, and full set nonmembership corresponding to scores of 5, 3, and 1, respectively. Further details can be found in Table 1.

**Table 1:** Condition assignment and calibration parameters for fsQCA

Condition	Assignment	Fully in	Max ambig	Fully out
Age	Years old	66 [90%]	46 [50%]	27 [10%]
Gender	1 = Female, 0 = Male	1	-	0
Marriage	1 = Married, 0 = Not married	1	-	0
Education	1 = Illiterate or semiliterate, 2 = Primary school, 3 = Junior high school, 4 = High school/technical secondary school/technical school/vocational high school, 5 = College degree/junior College, 6 = Master’s degree, 7 = Doctorate	6	4	2
Job income satisfaction	A scale of 1–5 ranging from very dissatisfied to very satisfied	5	3	1
General trust	1 = Yes, 0 = No	1	-	0
Interpersonal relationships	A scale of 0–10 ranging from lowest to highest	10 (90%)	7 (50%)	5 (10%)
General health	1 = Extremely healthy/very healthy/relatively healthy, 0 = Average/unhealthy	1	-	0

0/1 indicates crisp sets, and no calibration is needed

The sufficiency analysis begins with a "truth table", which includes all logically possible configurations of conditions and requires thresholds for case frequency and consistency level to be established manually. Typically, the frequency threshold is 1 or 2, but due to the large sample size of 17,475 observations, we set the frequency threshold for this analysis to 10 and the consistency level threshold to the commonly employed threshold of 0.8. The preconditions were then classified as either core or peripheral based on the following criteria: "Core conditions are those that are both parsimonious and intermediate solutions, while peripheral conditions are those that occur only in intermediate solutions" [64], and core conditions can be viewed as "decisive causal components" [65].

## Results

### Necessity analysis

In Table 2, we present the outcomes of our analysis of the conditions necessary for good general health. For all conditions, the states of presence and absence were included in the analysis of the necessary conditions. As the results shown in Table 2 demonstrate, it is not the case that the presence and absence of any condition is necessary for good general health (consistency < 0.90). The consistency of the structural social capital indicator "interpersonal relationships" was 0.508, and that of the cognitive social capital indicator "general trust" was 0.607; both values were less than 0.90. Of the other conditions, only the condition of "marriage" had a consistency result value of 0.803, which was the closest value to 0.90, thus suggesting that the state of marriage may be a condition that promotes the components of good general health.

**Table 2:** Analysis of the necessary conditions for good general health

Condition	Consistency	Coverage
Old	.431	.682
~ Old	.569	.844
Female	.455	.736
~ Female	.545	.793
Married	.803	.750
~ Married	.197	.837
Education	.314	.856
~ Education	.686	.731
Job income satisfaction	.648	.791
~ Job income satisfaction	.352	.723
General trust	.607	.795
~ General trust	.393	.725
Interpersonal relationships	.508	.786
~ Interpersonal relationships	.492	.746

The tilde “ ~ ” represents negation

### Sufficiency analysis

The subsequent phase aims to assess sufficiency. In general, 11 configurations were analysed that met the consistency requirement based on a consistency threshold of 0.8, as shown in Table 3. Based on the magnitude of the values for raw coverage and unique coverage, it can be concluded that configuration 3 (not being "old", not being "female" and the presence of "general trust") is most frequently associated with good general health.

**Table 3:**
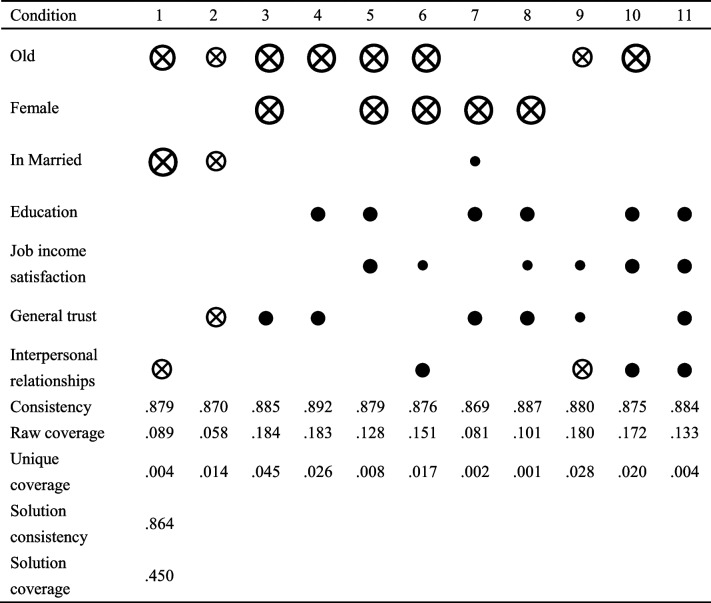
Analysis of the configurations for general health

Full black circles indicate the presence of a condition, and crossed open circles [“ ⊗ ”] indicate the absence [or negation] of that condition. Blank spaces indicate “do not care” [i.e., the condition score, whether high or low, is unimportant in that particular configuration with regard to the outcome]. Large circles suggest core or central conditions, whereas small circles indicate peripheral or contributing/complementary conditions

The two indicators of social capital chosen for this study, general trust and interpersonal relationships, are either part of the configuration or rated as "do not care", and neither one of these indicators nor a combination of the two can stand alone as a configuration that promotes good general health. In terms of the overall distribution, the two conditions of social capital are present in 10 of the 11 configurations, with all of them being core conditions of the "present" state in seven configurations (3,4,6,7,8,10,11). Individually, "general trust" appears in seven configurations, of which it appears as a core condition in five, while "interpersonal relationships" appears in five configurations, of which it appears as a core condition in three. The lowest number of additional conditions is 2, and the highest number of such conditions is 4. In this analysis, there are also configurations in which the social capital condition is absent (1,2,9), in which context "interpersonal relationships" is absent in configurations 1 and 9, and "general trust" is absent in configuration 2; they are all peripheral conditions in the corresponding configuration.

Among the other conditions, first, "old" is a fairly important condition, appearing in a total of eight configurations, six of which are core conditions; however, in all cases, these conditions appear as absences, indicating that age plays a significant role in health but that it must also be combined with other conditions. This condition is followed by "education" and "job income satisfaction", both of which appear in six configurations, with "education" appearing as the core condition in all six configurations and "job income satisfaction" appearing as the core condition in only three of the configurations. The next most important conditions are gender and marital status. The condition of "female" appears in all five configurations but in a state of absence, suggesting that not being "female" is more likely to lead to good general health outcome. The condition "marriage" appears in three configurations, in two as a noncore condition and in one as a core condition; however, in configuration 1, in which it is a core condition, it appears in a state of absence, and all three conditions in configuration 1 ("old", "marriage", and "interpersonal relationship") appear in a state of absence.

From a configuration perspective, the number of conditions in each configuration is 3 or 4, indicating that a combination of multiple conditions is required to achieve good general health outcomes. In both configurations 1 and 2, the state of the required condition is that of negation, and configurations are a combination of a condition from the social capital category with the "old" and "marriage” conditions. If the condition "marriage" is abolished, it is necessary to add the conditions "job income satisfaction" and "general trust" (configuration 9). In the conditions that appear in a state of presence, "education" appears in combination with either "job income satisfaction" or "general trust", and in configuration 11, "education", "job income satisfaction", and the two social capital conditions are combined, both appearing as core conditions.

## Discussion

This study aims to explore the relationship between social capital and general health from the new perspective of configuration. We employ the fsQCA approach to investigate publicly available data drawn from China, and we select conditions that have been widely validated, resulting in scientific and interesting results. We find that regardless of whether the indicators chosen to reflect social capital are present in this study, they are not necessary for good general health. Although these indicators are not necessary for individual analysis, they can be combined with other conditions to achieve good general health outcomes. In the configurations derived from the sufficiency analysis, the vast majority of social capital appears in a state of presence, suggesting that the presence of social capital promotes positive health outcomes. This finding is consistent with the majority of previous studies, which have concluded that there is a positive relationship between social capital and health [66–68]. However, we should also note that in two configurations, social capital is present in the state of negation, suggesting that lower social capital promotes good general health when combined with certain conditions, a result that is consistent with the conclusions of previous studies that have reported an inverse correlation [69, 70]. In addition, we find that social capital alone does not affect individual health and that whatever state it exhibits, it must be combined with other factors to achieve good overall health outcomes; furthermore, in some configurations, it is a "noncore" factor. This finding helps us understand the results of studies that have concluded that social capital is associated with health to a lesser extent or in a nonlinear way [71]. Furthermore, there is another configuration in which the condition for social capital does not appear, suggesting that in this configuration, there is no relationship between good general health and whether social capital is high or low, which may explain the findings of the rare studies that have concluded that social capital and health are unrelated [72]. Overall, the two conditions of social capital are indeed included among the outcomes that promote good general health.

Specifically, the configuration of the two negative states of social capital suggests that in cases of less social capital, either cognitive or structural, young people in nonmarital situations have better general health. Being unmarried, lower levels of interpersonal relationships, and low levels of trust in others can indicate a more "solitary" state, and it can be argued that young people feel healthier in the absence of the pressures of marriage and social activity. Some studies have found a curvilinear relationship between group membership and mental health: moderate participation is desirable, while too much or too little participation can have negative consequences [73]. In addition, some reviews have suggested that the mixed and negative health outcomes of the structural components of social capital might be the result of stress caused by an excessive burden stemming from the individual’s responsibilities [23]. Furthermore, we observe the simultaneous emergence of the two conditions of social capital, which is in line with Glanville et al.'s claim that "the effects of trust and social networks may be conditional on one another" [8]; nonetheless, both conditions must be complemented by high levels of education and income satisfaction to promote good general health. Better interpersonal relationships indicate relatively high levels of social interaction. According to the information dissemination pathway discussed above, health-related information is transmitted and diffused through social interaction, thus enabling individuals to obtain more health information, which in turn leads to more health information [74]. Trust in others facilitates the translation of this information into action, but this translation must be supported by a high income, which is consistent with dependency theory [75].

A very important variable is the "old" condition, showing that age is negatively related to health and has a great impact on health. Previous empirical studies of the relationship between social capital and health [11] have shown that age is an important sociodemographic variable, and the results also suggest a negative relationship with health. In a configuration study conducted using data from the European Social Survey, age did not have such a significant impact [1], which might imply, to some extent, that age is a more important factor with regard to the health of the Chinese population than to the health of the population in Europe, a point which requires our attention. The next two more important conditions are education and job income satisfaction, both of which appear in more than half of the configurations and both of which appear as core conditions. The presence of high levels of education and satisfaction either with or without the presence of social capital can lead to good general health. Higher levels of education tend to be positively related to work income, which leads to high-income satisfaction, and previous empirical studies have shown that education and income are positively related to health [76], a claim which is consistent with the results of this configuration analysis.

In our study, the condition of gender did not play the important role expected, with the results showing that the condition "female" was rated as "do not care" in more than half of the configurations. Despite the fact that numerous studies have reported gender differences in health [62, 77], our study suggests that other conditions may exist that weaken the effect of gender on health, such as education and job income satisfaction. Finally, the condition of marriage was shown to be less significant, and although a slight effect of being married on general health was observed, its presence was not necessary in more configurations.

### Limitations

Our study has certain limitations. First, although we cited many pertinent empirical studies to support our choice of indicators, due to the complexity and multifaceted nature of the two concepts of social capital and health, our indicators can reflect only limited aspects of social capital and health and are still unsuitable for further generalization. Second, due to issues pertaining to data access, we were unable to obtain information regarding the provinces in which the cases were located and thus did not analyse the differences in configuration across regions. Despite these limitations, this study provides an early example of the approach of using configuration analysis to study social capital and health in China, and we believe that the findings of this study may offer new ideas pertaining to the study of the correlation between social capital and health and contribute to the body of evidence on this topic in the Chinese context.

## Conclusion

This study revealed both a positive and a negative relationship between social capital and health, with cognitive social capital playing a greater role in the positive relationship than structural social capital. However, social capital is neither a necessary nor a sufficient condition for health on its own but must rather be combined with certain other conditions in different combinations to achieve good general health. Different people require different approaches to health; one pathway does not apply to all people, and there are a variety of ways in which the health of a specific individual can be promoted. In addition, our research shows that age, education, and income are components of various important combinations of conditions with regard to good general health.

## Data Availability

The datasets generated and/or analysed during the current study are available in the China Family Panel Studies repository, http://www.isss.pku.edu.cn/cfps/en/.

## Abbreviations

CFPS: China Family Panel Studies
fsQCA: Fuzzy-set qualitative comparative analysis
